# Recruitment of community‐dwelling oldest‐old African Americans for a study on the associations of depression and inflammation with cognitive decline

**DOI:** 10.1002/alz70860_102897

**Published:** 2025-12-23

**Authors:** Elizabeth Guerrero‐Berroa, Ruby S.C. Phillips, Kim Love, Vahram Haroutunian

**Affiliations:** ^1^ Lehman College, City University of New York, Bronx, NY, USA; ^2^ Icahn School of Medicine at Mount Sinai, New York, NY, USA; ^3^ K.R. Love Quantitative Consulting and Collaboration, Athens, GA, USA; ^4^ James J. Peters VA Medical Center, New York, NY, USA; ^5^ Alzheimer's Disease Research Center, Icahn School of Medicine at Mount Sinai, New York, NY, USA; ^6^ Nash Family Department of Neuroscience, Icahn School of Medicine at Mount Sinai, New York, NY, USA

## Abstract

**Background:**

African Americans are disproportionately burdened by dementia compared to other racial/ethnic groups in the U.S. However, factors contributing to this increased risk remain poorly understood, and especially in the oldest‐old segment of this population. A major concern raised by the cognitive aging literature is the underrepresentation and poor retention of African Americans in research, further limiting advancement in our understanding of risk and protective factors for dementia in this racial/ethnic group. The evidence on recruitment strategies of community‐dwelling oldest‐old African Americans for dementia research is almost non‐existent. We report on the methods, recruitment challenges, strategies, and modifications made to date, to a four‐year longitudinal study aimed at examining associations of depressive symptoms and inflammation with cognitive decline in initially non‐demented oldest‐old African Americans.

**Methods:**

Participants who self‐identified as Blacks/African Americans have been enrolled in this study. The initial enrollment age was 80+ years, and recruitment strategies included flyers, physician referrals, and educational presentations at senior centers.

**Results:**

In the first 28‐month recruitment period, 31 participants (mean age = 82.2 [*SD* = 4.8]; mean education = 14.3 [4.1]; 19.4%< high school; 87.1% females), have been enrolled. The most effective recruitment strategy was educational presentations at senior centers and churches (*n* = 16), which in turn, led to “snowball” sampling (*n* = 5). Inclusion of senior centers as an additional assessment location and provision of transportation have facilitated enrollment and retention. To date, of the 22 participants due for their 12‐month follow‐up visit, 17 (77.3%) have been assessed and two (9.1%) are to be scheduled.

**Conclusions:**

The relatively small number of oldest‐old Black/African American participants we have recruited in over two years showcases the significant difficulty in recruiting these participants. However, it is noteworthy that the retention rate of these participants show commitment to research despite their older age. Educational presentations at senior centers and churches should be ongoing to facilitate rapport and trust in the Black/African American community. Research enrollment and retention is possible by building strong connections with community leaders, such as directors of senior centers. Recruitment of Blacks/African Americans requires considerable time and effort, for which investigators should consider allocating sufficient funding dedicated to recruitment.

